# Synchrotron X-ray microtransections: a non invasive approach for epileptic seizures arising from eloquent cortical areas

**DOI:** 10.1038/srep27250

**Published:** 2016-06-06

**Authors:** B. Pouyatos, C. Nemoz, T. Chabrol, M. Potez, E. Bräuer, L. Renaud, K. Pernet-Gallay, F. Estève, O. David, P. Kahane, J. A. Laissue, A. Depaulis, R. Serduc

**Affiliations:** 1 Inserm, U1216, F-38000 Grenoble, France; 2 Univ. Grenoble Alpes, Grenoble Institut des Neurosciences, GIN, F-38000 Grenoble, France; 3 Synapcell S.A.S – Bâtiment Biopolis – 5 avenue du Grand Sablon, La Tronche, France; 4 ESRF, Grenoble, France; 5 Univ. Grenoble Alpes, EA RSRM, F-38000 Grenoble, France; 6 CNRS; CE2F PRIM UMS3537; Marseille, France; 7 Aix Marseille Université; Centre d’Exploration Fonctionnelle et de Formation; France; 8 CHU Grenoble Alpes, F-38000 Grenoble, France; 9 University of Bern, Switzerland

## Abstract

Synchrotron-generated X-ray (SRX) microbeams deposit high radiation doses to submillimetric targets whilst minimizing irradiation of neighboring healthy tissue. We developed a new radiosurgical method which demonstrably transects cortical brain tissue without affecting adjacent regions. We made such image-guided SRX microtransections in the left somatosensory cortex in a rat model of generalized epilepsy using high radiation doses (820 Gy) in thin (200 μm) parallel slices of tissue. This procedure, targeting the brain volume from which seizures arose, altered the abnormal neuronal activities for at least 9 weeks, as evidenced by a decrease of seizure power and coherence between tissue slices in comparison to the contralateral cortex. The brain tissue located between transections stayed histologically normal, while the irradiated micro-slices remained devoid of myelin and neurons two months after irradiation. This pre-clinical proof of concept highlights the translational potential of non-invasive SRX transections for treating epilepsies that are not eligible for resective surgery.

Surgical resections for intractable epilepsies have reached impressive success rates in recent years^1^,^2^. However, the management of seizures arising from eloquent cortical regions remains challenging due to high rates (up to 50%) of post-operative neurological deficits^3^,^4^,^5^,^6^. Non-resective procedures such as vagus nerve stimulation^7^, scheduled deep brain stimulation^8^ or closed-loop neurostimulation^9^, did not achieve seizure freedom in most cases. Multiple Subpial Transection (MST), developed by Morrell *et al.*^10^, is currently the most effective surgical technique applicable to eloquent cortex. Transections made with a special surgical knife at intervals of 5 mm interrupt horizontal intracortical fibers, while preserving vertical fibers. The procedure is based upon experimental evidence indicating that epileptogenic discharges require substantial side-to-side or horizontal interaction of cortical neurons^11^, whereas major functional properties of cortical tissue rely greatly on vertical fiber connections of the columnar units^12^. MST reduces synchronized discharges from the epileptic focus and limits their spread without impairing the function of the transected cortex^13^. However, MST is an invasive procedure that only reaches moderate rates of seizure freedom because of the limited access to deep cortical tissue/sulci^14^,^15^ and the risk of post-operative deficits, such as brain edema, infections, and bleeding^16^. Radiosurgery is an attractive alternative to surgical MST but radio-transections are currently unachievable with conventional radiosurgical devices and/or mega-voltage machines since their lateral dose profile does not provide the required sharp dose demarcations between irradiated and non-irradiated tissue slices^17^,^18^. Further, the transection procedure requires focused delivery of hectogray doses^19^, currently only achievable at 3^rd^ generation synchrotron facilities.

The assessment of the efficacy of synchrotron radiation for seizure management in rodents has been studied during the last few years using different methods and irradiation configurations^20^,^21^,^22^,^23^. The purpose of the present study was to perform a novel method of non-invasive cortical transections in epileptic rats using synchrotron x-rays (SRX) and to quantify the induced changes of normal and pathological electrical activities. To achieve this, we developed an innovative technique that focally cuts tissue within the cortex with micrometer precision. The synchrotron light was shaped into 50 μm-wide parallel microbeams characterized by extremely steep dose-gradients (about 200 hundred times steeper than conventional X-rays^23^. We arranged them spatially to achieve microtransections that aimed at “disconnecting” tissue slices from each other. The sensorimotor cortex of the GAERS rat, a validated model of generalized epilepsy^21^,^24^,^25^, was chosen as the target because it is non-resectable without functional deficit and produce frequent naturally-occurring highly-synchronized oscillations, *i.e*. Spike-Wave Discharges (SWDs). GAERS were chronically implanted with two symmetrical/longitudinal rows of five depth electrodes in both cortices (Fig. 1c). We chose to apply the transections mid-way between the electrodes of the left cortex only, to maintain the contralateral cortex as a control of the seizure stability across time. More precisely, four 200 μm-wide, 2 mm spaced, transections were delivered using submillimetric image-guidance (*23*) (Fig. 1a–d). Each transection was made by four 50 μm-wide and 2 mm-high microbeams with an entrance dose of 800 Gy each, sequentially delivered to the target through ports angled at 0°, 45°, 90° and −45°; Fig. 1a,b). Between each irradiation port, the animal was shifted laterally by 50 μm. The interlacement of the four microbeams resulted in a 200 μm wide prismatic octahedron with a diameter of 2 mm, exposed to 820 Gy (dose profile: Fig. 1e). This procedure was repeated 4 times at different antero-posterior coordinates.

## Results

### Synchrotron-generated microbeam induced sharp transections in rat brain

**Figure 1:**
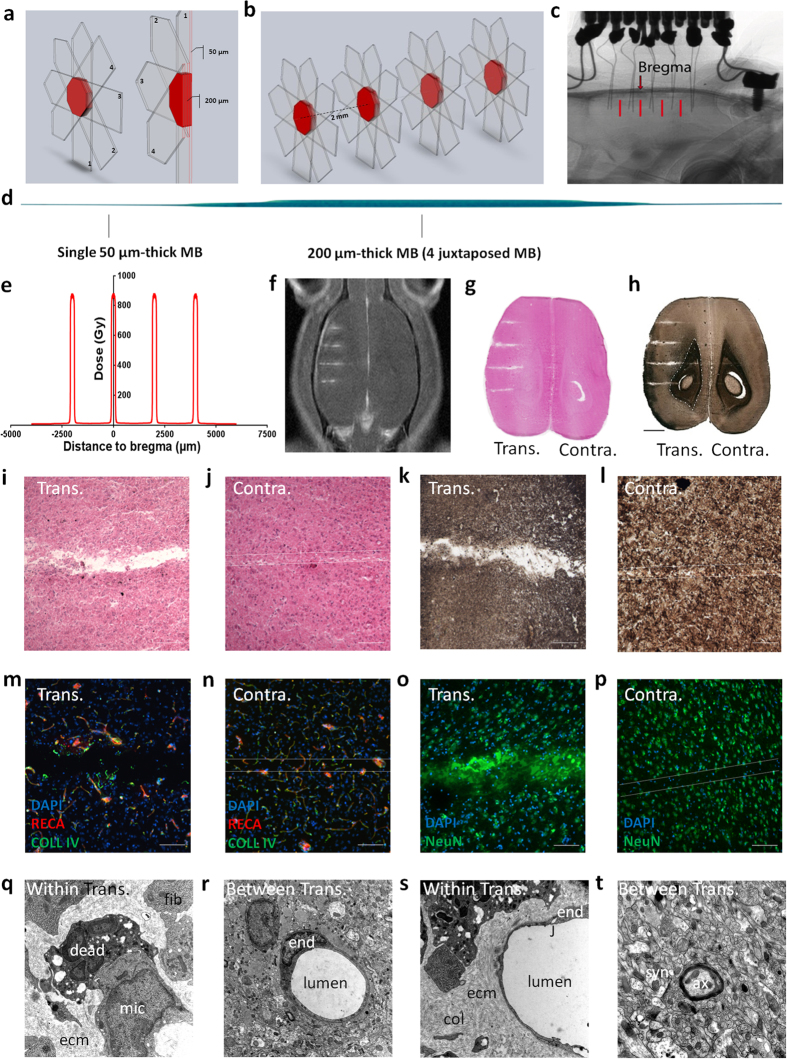
Synchrotron X-ray transections. Irradiation parameters and tissular effects. (**a**,**b**) 3D Microbeam interlacement; transparent arrows denote the trajectory of each microbeam (MB). Numbers represent the sequence of their delivery. The interlaced volume (transection) is shown in red. (**c**) X-ray image of a GAERS brain with two rows of 5 implanted electrodes. Transections are indicated by red bars. (**d)** The scan of a Gafchromic™ film shows 4 microbeams, interlaced to create a 200 μm wide, octahedral irradiated transection. (**e)** Radiation dose deposition profile for the 4 transections. Estimated peak dose in the transection 820 Gy, entrance dose of a single microbeam 800 Gy. (**f)** Gadolinium contrasted T_1_ MR image of a rat after 4 transections. (**g–t)** Horizontal brain sections 2 months post-irradiation, haematoxylin & eosin (**g**), myelin stain (**h**); scale: 2 mm, white/grey matter limit is represented as a dashed line. Microphotographs of cortical transections (**i**,**k**) show a tissue gap (**i**), and myelin loss (**k**), while the path of a single microbeam in the contralateral hemisphere shows only loss of cell nuclei (**j**,**l**; scale: 200 μm). Immunolabelling of brain vasculature (RECA and Type IV Collagen, **m**,**n**) and neurons (NeuN, **o**,**p**) in transected (**m**,**o**) and contralateral (**n**,**p**) hemispheres (scale: 50 μm). Ultrastructural changes, as described in the text, within (**q**,**s**) and between transections (**r**,**t**). Within transection (**q**) extracellular space is filled with extra cellular matrix (ecm) secreted by fibroblasts (fib) and phagocytosis of dead cells by microglial cells (mic) are observed. Capillaries were intact (**s**) and the junctions (**j**) of endothelial cells (end) are closed but some vacuoles can be observed (**j**). Extracellular matrix and collagen fibrils (col) are observed around the capillary. Between transections (**r**,**t**), the endothelial cells of the capillaries were not damaged **(r)**, nor the surrounding tissue, including synapses (syn) and axons (ax) (**t**). (TEM, magnification 12,000).

Figure 1f–t depicts several aspects of the transection-induced damage in the brain two months after irradiation. Tissue necrosis was confined to the transection sites irradiated by the four interlaced microbeam paths. No tissue damages were observed between the transections. MR T_1_w images examination showed Gd-DOTA leakage confined to the 200 μm-wide transection sites while no diffusion was detected in the cortex of the contralateral hemisphere within the paths of non-interlaced 50 μm-wide microbeams (Fig. 1f). Brain sections (Fig. 1g,h) revealed tissue disintegration resulting in a sharp interruption of normal connections such as myelin sheaths within the transection sites (Fig. 1i,k). Conversely, the normal tissue structure, as well as myelin sheaths, were preserved in the paths of microbeams in the cortex of the contralateral hemisphere (Fig. 1j,l). Blood vessels were only interrupted at the four transections sites, but never in slices irradiated by a single microbeam (Fig. 1m,n). Neurons, astrocytes and oligodendrocytes were missing in both 200 μm- or 50 μm-wide irradiated tissue slices (Fig. 1o,p). Ultrastructurally, the neuropile was clearly damaged within the transection sites (Fig. 1q,s) while tissues located in between appeared normal (Fig. 1s,t). The transection gaps were filled with fibroblasts containing a developed rough endoplasmic reticulum and with extracellular matrix (Fig. 1q), including collagen fibrils. Edema was observed around capillaries near the transections, (Fig. 1s), but not in interjacent tissues (Fig. 1r). Numerous microglial cells were found in the transection sites, with phagocytosed neuronal and other cellular debris (Fig. 1q).

### Synchrotron-generated transection reduces SWD power and coherence

**Figure 2:**
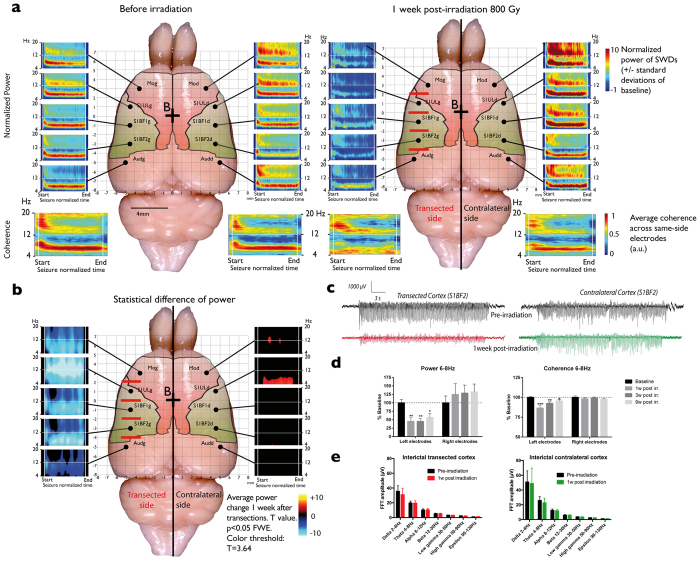
Pre- and post-irradiation records and statistics of seizures, seizure power, coherence and background signals. (**a)** Average (n = 4) normalized time/frequency maps of seizure power (time rescaled: start = 0; end = 1) recorded from the 10 electrodes (black points), before (left panel) and 1 week post-irradiation (right panel). The 4 lower maps represent the average coherence computed from the left and the right electrodes. (**b)** Difference of power between pre- and post-irradiation seizures: the t values are color- coded. Mediolateral coordinates are displayed by the grid overlying the brain. Time/frequency maps are normalized against the background signal preceding the seizures (duration: 5% of the seizure length). Red bars indicate the location of the transections. (**c)** Example of seizures recorded from the two symmetrical electrodes implanted in the transected (left) and contralateral (right) somatosensory cortex barrel fields (S1BF2) before (upper grey seizures) and 1 week after (lower red and green seizures) the transections. (**d)** Quantification of the mean seizure normalized power (left panel) and coherence (right panel) for the 3 central left and right electrodes before irradiation and 1, 3, 9 weeks post-irradiation. Data are expressed as percent of the baseline power/coherence of the 6–8 Hz band (*p < 0.05; **p < 0.01; Two-way repeated measure Anova, Sidak post-hoc). (**e**) Average fast-Fourier transforms (FFT) of background signals recorded from the left (left panel) and right electrodes (right panel) before (black histograms) and 1 week following irradiation (colored histograms). FFT amplitudes are displayed as frequency bands (delta, theta, alpha, beta, low gamma, high gamma and epsilon). There was no significant statistical differences between pre- and post-irradiation for any band (Two-way repeated measure Anova, p > 0.05).

Although microbeam irradiations did induce a slight decrease in the number and duration of SWDs one week after irradiation (Table 1), values returned to baseline levels at subsequent post-irradiation times. This lack of effect of unilateral irradiation on seizure burden is in accordance with previous reports showing that bilateral manipulations are necessary to change the occurrence of seizures in this model^26^. However, from one week after transection, the power of the SWDs (6–8 Hz) recorded from the left electrodes in the irradiated left hemisphere was significantly reduced (Fig. 2a–d, p < 0.01, Two-way ANOVA, Bonferroni’s post hoc test). By contrast, the single microbeam exposure had no effect on the power of SWDs recorded from the right cortex. Transections between electrodes of the irradiated cortex also induced a unilateral decrease of coherence between the signals during SWDs (Fig. 2a,d, p < 0.01, Two-way ANOVA, Bonferroni’s post hoc test), *i.e.* a decrease of wide-scale cortical network synchronization. The latter remained significantly different from baseline to 9 weeks after transections (Fig. 2d). These observations suggest that horizontal communication between the brain “slices” was significantly altered by the transections. By contrast, the background (interictal) EEG signal remained unaltered by the procedure (Fig. 2e), Indeed, the power of all frequency bands was unchanged by the irradiation (p > 0.05, Two-way ANOVA, Bonferroni’s post hoc test), suggesting that the physiological activity of neuronal networks within the non-irradiated tissue slices remained intact.

## Discussion

Radiosurgeries for epilepsies arising from eloquent areas are currently inapplicable because of the excessive lateral dose falloff distance that limits the radiation dose safely deliverable to the target. The physical properties of synchrotron light, *i.e*., its quasi non-divergence (3.3 × 0.1 mrad^2^), high flux (2.10^14^ ph.s^−1^/0.1% BW/100 mA/mrad) and kilovoltage energies (50–350 keV), allow unparalleled dose-gradients in tissues: SRX exhibit a 90% to 10% lateral dose falloff of 50 μm *versus* 9 mm for the Perfexion gamma knife^23^.

We recently showed that high-dose interlaced SRX delivered bilaterally to the somatosensory cortices of GAERS rats (6 × 2 × 2.5 mm^3^) prevented the targeted structure from initiating SWDs and decreased seizure duration in the rest of the cortex and the thalamus^21^. Presently, we reduced the total irradiated volume by a factor 8 in order to limit the risk of side effects and to permit the safe application of the technique to eloquent cortices. The transections disconnected neighboring cortical tissue slices from each other both physically and neuro-physiologically. Our LFP recordings in interjacent tissue slices showed that, between seizures, the background EEG signal remained unaltered by the irradiations (Fig. 2e), as well as the ictal/interictal signal recorded from the contralateral cortex, which only received single 50 μm-wide microbeams. This correlates with the absence of histological (Fig. 1j,l,n,p) and ultrastructural changes (Fig. 1r,q) in non-transected sites.

Our procedure meets several desirable features for clinical application: i) it is non-invasive; ii) the reductions of SWD’s power and coherence can be observed as soon as one week post-irradiation, a delay that is unusual in current radiosurgery; iii) the tissue between transections appears to be histologically unaltered and, iv) the power of epileptic activities are reduced during at least 9 weeks. The technical developments and safety requirements for SRX therapy are already fulfilled for the current Phase 1-2 clinical trials for brain cancer patients (#2010-A00773-36). Current pre-clinical tests in non-human primates are paving the way towards a transfer of SRX microtransections to the clinic by giving evidence on another epilepsy model (tetanus toxin induced cortical epilepsy) that SRX is relevant for an alternative epilepsy management.

## Methods

### Animals

All experiments were approved by local Ethical Committees (ETHAX and Grenoble Neuroscience Institute, licenses 380324 and A3818510002) and European Union guidelines (directive 86/609/EEC). All experiments were performed in accordance with relevant guidelines and regulations and every precaution was taken to minimize stress and the number of animals used in this study. This experiment has been performed in the GAERS model of absence epilepsy because we have (i) described the epileptic process in this model, (ii) already performed MRT exposures in this model and (iii) described for the first time the reduction of seizures by irradiating the initiating focus. The GAERS model provides an excellent stability in the number, duration and power of its numerous spontaneous seizures^27^, which allows a precise quantification of the anti-epileptic effect of the procedure. GAERS model allowed us to evaluate the connectivity changes between slices and to demonstrate the efficiency and non-invasiveness of synchrotron transections in epileptic focus parcelization. Four adult female GAERS were implanted with electrodes, irradiated in the S1Cx by IntMRT and EEG recorded at different delays after exposures. Five additional rats were irradiated, MR imaged and culled for microscopic (histology n = 3; transmission electronic microscopy n = 2) brain examinations.

### Multisites local field potential recordings in freely moving rats

Functional experiments were performed in four adult female GAERS. They were anesthetized (100 mg/kg ketamine, i.p. plus 10 mg/kg xylazine, i.p.) and placed into a stereotaxic frame. Two symmetrical rows of 5 stainless steel wire electrodes (Ø: 0.125 mm, polyester-isolated) were bilaterally implanted at different sites in the motor, somatosensory and auditory cortices with bregma as the zero reference^28^. A reference was placed over the olfactory bulbs and a ground electrode over the cerebellum. All electrodes were fixed to the skull with cyanoacrylate and dental acrylic cement and connected to two female connectors (one for each hemisphere). One and three weeks after surgery rats were placed in a PMMA box, where they could freely move and were recorded for a minimum of 1 h. Local field potentials (LFPs) were amplified through two miniature headstage preamplifier (MPA-8-I, 8-channel single-ended amplifier with one common subtracting input for an indifferent electrode; voltage gain, x10; frequency band, DC 5 kHz; Multi Channel Systems, Reutlingen, Germany) connected to a 32-channel programmable gain amplifier (PGA-32; voltage gain, x200; Multi Channel Systems) and sampled at 5 kHz (16 bit ADC). Recordings were collected on a personal computer via a CED interface (Cambridge Electronic Design, Cambridge, UK) using the Spike 2 software.

### Signal analyses

We performed time-frequency analysis of SWDs power and coherence, using Welch’s periodogram method as implemented in pwelch.m Matlab routine, between 4 and 20 Hz with 0.1 Hz steps on cortical signals collected in freely moving GAERS using a time-window of 4 s duration positioned every 100 ms. The first and last spikes of each SWD were used to define its onset (start) and termination (end), respectively. The time-window of analysis of each discharge was adapted to the duration of the discharge and started before its onset (pre SWD duration chosen as 10% of SWD duration) and terminated after SWD offset (post SWD duration chosen as 10% of SWD duration). Power and coherence maps were normalized in amplitude using a standard procedure: for each frequency, the mean of power/coherence during the baseline (pre SWD period) was subtracted from the power/coherence during the whole duration of the event and then demeaned power/coherence were divided by their standard deviation computed during the baseline. Finally, the time axis of the time-frequency chart was linearly rescaled to realign the start and end of SWDs across recordings in order to allow statistical analyses of signals in the time-frequency plane over all the SWD duration. Inference on normalized and rescaled 2D time-frequency maps were performed for each channel separately by comparing pre- and post-irradiation SWDs using the random field theory to control for multiple comparisons performed in the time and frequency directions^29^. To conform to the assumptions of random field theory, images were smoothed by convoluting a Gaussian kernel in both time (5% of seizure duration FWHM) and frequency (5 Hz FWHM). In addition, this step blurs any effects that are focal in the time and/or frequency dimensions, ensuring a better overlap between animals and seizures. A paired two-sample t-test comparing pre- and post-irradiation SWDs was used to characterize differences in seizure power. In all cases, a fixed-effect analysis was assumed by pooling together animals and seizures.

Interictal signal portions were epochs of 2–10 s before the SWDs considered in this study. Fast-Fourier transforms (FFT) were computed and the FFT amplitude was averaged in each frequency band.

Signal analyses were done using an in-house Matlab toolbox for all Morlet wavelet transforms based on routines provided by the SPM8 and Fieldtrip softwares.

### Statistical analyses

In order to perform statistical analyses on the time-course of the power and coherence of SWDs following irradiation, only the 3 central electrodes of each hemisphere were considered to restrain the analysis to the brain “slices” surrounded by the transections. Given that most of the power and coherence of the SWDs was carried by the 6–8 Hz frequency-band, it was chosen to quantify the irradiation effect on the SWDs. Power and coherence from these 3 central electrodes from each hemisphere were then averaged at each time point and expressed percent of baseline power/coherence. Repeated-measure 2-way ANOVAs were applied to these average values with “time” (baseline, 1, 3 and 9 week post-irradiation) as between factor and “cortical side” (left hemisphere, right hemisphere) as within factor. Post-hoc Bonferronni’s tests were used to compare the each post-irradiation value to baseline levels. The power of the interictal frequency bands before and after irradiation were compared using 2-way repeated measure ANOVAs with “time” (pre- and post-irradiation) as between factor and “frequency band” as within factor. Post-hoc Bonferronni’s tests were then applied to compare post-irradiation value to baseline level for each frequency band. P < 0.05 was considered as the significance threshold for all analyses.

### Beam alignment and IntMRT

MRT was performed at ID17, the biomedical beamline of the European Synchrotron Radiation Facility (Grenoble, France). X-rays were produced from a 1.6T wiggler located at 43 m from the sample. The white beam was filtered with Be (0.5 mm), C (1.5 mm), Al (1.5 mm) and Cu (1.0 mm) resulting in a spectrum extending from 50 to 350 keV (median energy: 107 keV). The dose rate in air at the animal surface was approximately 16,000 Gy.s^−1^. Beam height and width was defined using tungsten slits and quasi-laminar microbeams were produced with the ESRF multislit collimator^30^. We performed IntMRT as previously described in detail in^23^. Anaesthetized GAERS were positioned on a rotation stage installed above a goniometer and X-ray projections images (35–60 keV) of the rat head and electrodes were acquired to delineate the irradiation fields defined from the *bregma*. A schematic representation of the irradiation configuration is shown in Fig. 1a,b. Each transection was performed by one single 50 μm-thick/2000 μm-wide MB entering the brain through 4 irradiation ports separated by a 45° angle and a 50 μm step, both applied in the y-axis, thus resulting in a localized solid dose deposition in a 200 μm-thick/2000 μm-diameter quasi-cylindrical target. Four such transections were realized between the five left LFP electrodes −4.5 mm laterally from the at midline at antero-posterior coordinates +2 mm, 0 mm, −2 mm and −4 mm (red lines on Figs 1c and 2a,b). The resulting dose prints for one microbeam (50 μm-wide) and the radiation target (200 μm, juxtaposed microbeams) are shown on a gafchromic film on Fig. 1d. We assessed volumic dose in a rat head phantom by using PENELOPE-2006 Monte Carlo code^31^,^32^ and the profile obtained through the rat cortex at the center axis of the radiation targets is shown on Fig. 1e. The MB entrance doses were 800 Gy for individual microbeams resulting in an 820 Gy radiation dose at the targeted sites. The dose built-up phenomenon is described in ref. 23.

### Magnetic resonance (MR) imaging

Two months after irradiation, we obtained 7T-MRI (Bruker Biospec Avance III) in all rats. We characterized brain vessel permeability using a T1w sequence (TurboRARE. TR: 1300 ms, effective TE: 7.7 ms, FOV: 30 × 30 mm, matrix: 256 × 256, slice thickness: 0.5 mm) acquired ~5 min after intravenous injection of Gadolinium (200 mmol.kg^−1^, Dotarem, Guerbet).

### (Immuno)histological procedures

At the end of the MR examination, rats were decapitated, brains excised and frozen. HE staining was performed on 20 μm-thick horizontal brain sections. The myelin staining was performed according to a protocol modified from Gallyas^33^. Before staining, the sections were fixed with PFA 4% for 24 hours. After incubation in a silver nitrate solution for 1 h, sections were washed in 0.5% acetic acid solution. They were then left in a developer solution freshly prepared as in the original staining procedure, washed in 0.5% acetic acid solution and differentiated in 0.2% potassium ferrocyanide. This step was repeated three times before final fixation in thiosulfate followed by dehydration and mounting. Immunohistological procedures for Type-IV Collagen, Rat Endothelial cell Antigen (RECA) and NeuN are detailed in ref. 23.

### Transmission electronic microscopy

To investigate the ultrastructure of the lesion, 2 rat brains were fixed by perfusion with 2% paraformaldehyde and 0.2% glutaraldehyde in 0.1 M phosphate buffer pH 7.2. Cortex of the site of the lesion was dissected and cut in 1 mm^3^ pieces before being quickly immersed in fixative (2% PFA and 2% glutaraldehyde in 0.1 M cacodylate buffer) during 2 hours. Samples were then embedded in an epoxy resin. For this purpose, samples were washed, post-fixed with 1% osmium tetroxyde in cacodylate buffer, stained in 1% uranyl acetate in water pH 4 during 1 hour in the dark and dehydrated through graded ethanol series before being embedded in epoxy resin (Flukka) that was polymerized during 3 days at 60 °C. Ultrathin sections of the sample (60 nm) were cut with an ultra-microtome (Leica ultracut). Sections were then stained in uranyl acetate and lead citrate before being viewed under a transmission electron microscope at 80 kV (JEOL 1200EX). Images were taken at 12,000 and 50,000 magnifications with a digital camera (Veleta, Olympus) and morphometry was done with iTEM software (Soft Imaging System). Experiments were performed at the Electron Microscopy facility of Grenoble (MEC).

## Additional Information

**How to cite this article**: Pouyatos, B. *et al.* Synchrotron X-ray microtransections: a non invasive approach for epileptic seizures arising from eloquent cortical areas. *Sci. Rep.* **6**, 27250; doi: 10.1038/srep27250 (2016).

## Figures and Tables

**Table 1:** Number of SWDs and average seizure durations measured at different time points after treatment.

	Baseline	1 week post irr.	3 weeks post irr.	9 weeks post irr.
Number of SWDs/hour	62.2 ± 8.1	35.2 ± 2.8	50.2 ± 7.5	47.2 ± 3.9
Average seizure duration (s)	24.8 ± 2.7	17.2 ± 2.6	20.96 ± 2.8	19.8 ± 2.0
